# Personal

**Published:** 1904-03

**Authors:** 


### PERSONAL.

Dr. J. N. McCormack, of Bowling Green, Ky., secretary of the Kentucky state board of health and chairman of the committee of organisation of the American Medical Association, attended the ninety-eight annual meeting of the Medical Society of the State of New York at Albany, January 26-28, 1904. Dr. McCormack met many old friends and made hosts of new ones on that occasion, all of whom were glad to shake his cordial hand and listen to his charming conversation. His speech at the annual dinner was one of his best efforts and received the generous plaudits of the banqueters.

---

Dr. Nelson W. Wilson, of Buffalo, has been appointed assistant to Dr. Park in the department of genitourinary surgery at Buffalo General Hospital.

---

Dr. Henry N. Miller, of Lancaster, N. Y., has removed to Buffalo and established his office and residence at No. 381 Hudson street. He is a brother of Dr. J. G. Miller, the well-known physician at Lancaster.

Dr. F. H. Millener, of Buffalo, addressed the Buffalo Society of Natural Sciences on Friday evening, February 12, 1904, his subject being The induction coil and its resulting phenomena,—the *x*-ray and wireless telegraphy. The lecture was illustrated by apparatus a portion of which Dr. Millener, himself, constructed. The address was well received by a large audience.

---

Dr. Dewitt G. Wilcox, of Buffalo, was elected secretary of the Homeopathic Medical Society of the State of New York at its annual meeting held at Albany, February 11, 1904.

---

Dr. John H. Pryor, of Buffalo, has passed a successful examination before the State Civil Service Commission, thereby qualifying for the position of superintendent of the state hospital for consumptives at Ray Brook. Dr. Pryor is practically the founder of the institute, having first proposed its establishment in a paper read some years ago before the Medical Society of the State of New York. He afterward prompted the introduction of a bill into the legislature establishing the institution and led in the contest, through its several stages, until it was finally enacted into law.

---

Dr. William R. Pryor, of New York, read a paper before the section of obstetrics and gynecology, Buffalo Academy of Medicine, Tuesday evening, February 23, 1904, entitled, The technique of a radical abdominal operation for carcinoma of the uterus. Dr. M. D. Mann entertained Dr. Pryor and prominent local physicians at dinner at his residence, 31 Allen street, just previous to the meeting.

